# Treatment of Rivaroxaban versus Aspirin for Non-disabling Cerebrovascular Events (TRACE): study protocol for a randomized controlled trial

**DOI:** 10.1186/s12883-015-0453-7

**Published:** 2015-10-12

**Authors:** Fang Yang, Wenrui Jiang, Ya Bai, Junliang Han, Xuedong Liu, Guangyun Zhang, Gang Zhao

**Affiliations:** Department of Neurology, Xijing Hospital, No. 15 West Changle Road, Xi’an, 710032 China; Emergency Department, Xijing Hospital, No. 15 West Changle Road, Xi’an, 710032 China

**Keywords:** Acute minor ischemic stroke, Transient ischemic attack, Rivaroxaban, Aspirin, Anticoagulation

## Abstract

**Background:**

Transient ischemic attack (TIA) or minor ischemic stroke represents the largest group of cerebrovascular disease, and those patients have a high risk of early recurrent stroke. Over decades, anticoagulation therapy has been used prudently in them for likely increasing the risk of intra-/extra-cranial hemorrhagic complications. However, recently rivaroxaban, a new oral anticoagulant, is proved to be as effective as traditional anticoagulants, while carrying significantly less risk of intracranial hemorrhage. Therefore, we assumed that patients may benefit from rivaroxaban if treated soon after TIA or minor stroke, and designed this adequately powered randomized study, TRACE.

**Methods and design:**

The *Treatment of Rivaroxaban* versus *Aspirin in Non-disabling Cerebrovascular Events* (TRACE) study is a randomized, double-blind clinical trial with a target enrollment of 4400 patients. A 14-days regimen of rivaroxaban 10 mg daily or a 14-days regimen of aspirin 100 mg daily will be administrated to randomized participants with acute TIA or minor stroke, defined as National Institute of Health Stroke Scale scores ≤3. The primary efficacy end point is percentage of patients with any stroke (ischemic or hemorrhage) at 14 days. Study visits will be performed at the day of randomization, day 14 and day 90.

**Discussion:**

Even though the new oral anticoagulants seem to be both safe and effective, few clinical trials have been carried out to test their effect on non-disabling cerebrovascular events. Treatment with rivaroxaban may prevent more cerebrovascular events with an acceptable risk profile after TIA or minor stroke, compared with aspirin, thus helping to improve the outcome of the disease.

**Trial registration:**

No. NCT01923818

## Background

Transient ischemic attack (TIA) and acute minor ischemic stroke (MIS), defined as a National Institutes of Health Stroke Scale (NIHSS) score ≤3 [1], represent the largest group of patients with cerebrovascular disease. A total of 150,000 suspected TIAs and MIS are referred to secondary care for assessment and investigation in England alone each year [2], and approximately 300,000 TIAs are diagnosed each year in the United States [3]. Though TIA and MIS have commonly been referred as non-disabling cerebrovascular events, they often prognosticated a high risk of recurrent disabling stroke. Previous studies reported that [4, 5] the risks of subsequent stroke were as high as 10.5–18.5 % at 90 days after TIA or MIS, over a half of which occurred in the first week. Some even believed that the recurrent stroke risk was likely to be much higher than commonly thought, about one third patients with ischemic stroke have had an earlier TIA or MIS [6].

Thrombylisis therapy, antiplatelet therapy and anticoagulant therapy play important roles in treatments of ischemic cerebrovascular disease. Intravenous tissue plasminogen activator (t-PA) was identified as the only pharmacological treatment for acute ischemic stroke approved by the US FDA in 1996 [7], however, it usually excluded the MIS patients [8]. About one third of those, who hadn’t accepted intravenous t-PA treatment since stroke symptoms were mild on hospital arrival, would have a poor final stroke outcome [9, 10].

For long time, aspirin has been considered as the standard antiplatelet therapy for ischemic cerebrovascular diseases. Two large clinical trials indicated that over 2–4 weeks treatment with aspirin after acute ischemic stroke reduced the risk of recurrent ischemic stroke by 30 % with a small increase in intracranial hemorrhage [11, 12]. Previous trials [13, 14] on secondary prevention of noncardiogenic ischemic stroke had reported a combination of clopidogrel and aspirin had no further benefit compared with aspirin only. However recently, CHANCE study [15] excitingly reported that, not associated with increased hemorrhage events as compared with aspirin monotherapy, dual antiplatelet therapy (clopidogrel and aspirin) reduced the risk of recurrent stroke in patients with TIA and acute MIS. The results shed a light on exploiting possibly more effective medical therapy for patients suffered non-disabling cerebrovascular diseases.

Over 50 years, anticoagulants have been used to treat patients with acute ischemic stroke for preventing early recurrent stroke and improving neurological outcomes with the cited reasons including: (1) to halt neurological worsening; (2) to prevent early recurrent embolization; (3) to improve neurological outcomes. Although, traditional anticoagulant has showed effectiveness of recurrent stroke prevention, its use was very limited in patients with ischemic cerebrovascular events because of higher incidence of bleeding events [16–19]. Proper treatment for non-disabling cerebrovascular events should be effective, as well as safe and convenient. Recently, new oral anticoagulants have received extensive attentions because of the following advantages, 1) directly targeting the coagulation cascade with rapid onset/offset of action; 2) fewer side effects (especially lower rates of major hemorrhage); 3) lower risks for drug-drug interactions; 4) more predictable responses [20]. One of the new anticoagulants, rivaroxaban, a direct factor Xa inhibitor, has been approved in US for prevention of deep-vein thrombosis (DVT) and venous thromboembolism (VTE) in surgery and for prophylaxis of the risk of stroke in people with abnormal heart rhythm (non-valvular atrial fibrillation) at 2011, and for treatment of DVT and pulmonary embolism at 2012. Recent published trials [21, 22] confirmed that rivaroxaban reduced the mortality of severe cardiovascular events and improved outcome with less fatal bleeding in the patients with atrial fibrillation and in the secondary prevention of patients with acute coronary syndrome. Moreover, rivaroxaban showed the best cost-effectiveness of stroke prevention in patients with nonvalvular atrial fibrillation within the 3 novel oral anticoagulants, i.e. apixaban, dabigatran and rivaroxaban [23].

Till now, no specific acute therapy is available for the vast majority of patients with acute non-disabling cerebrovascular events other than antiplatelet therapy [15]. This new anticoagulant agent, rivaroxaban could be a more effective, as well as safe and convenient option. Therefore, in this study we will enroll Chinese patients with TIA and acute MIS, who would show particularly high risk for recurrent ischemia, low risk for hemorrhage and relatively stable systematic conditions after cerebrovascular events onset. And they will be randomly treated with rivaroxaban (10 mg daily) or aspirin (100 mg daily) to assess the safety and efficacy of the new medication.

### Study objective

The Treatment of Rivaroxaban versus Aspirin in Non-disabling Cerebrovascular Events (TRACE) study (www.clinicaltrials.gov identifier NCT01923818) is a randomized, double-blind, multicenter, controlled clinical trial in 4400 Chinese patients with acute TIA or minor stroke. The primary objective of this trial is to determine whether rivaroxaban is safe, when added to standard care, and can reduce the risk of any stroke (both ischemic and hemorrhagic) when initiated within 24 h of symptom onset in high-risk patients with TIA or MIS.

### Study participants

Totally 4400 patients will be recruited through 60 emergency departments of general hospitals in China. Two subtypes of patients would be enrolled: I, acute non-disabling ischemic stroke (<24 h of symptoms onset); II, acute TIA (<24 h of symptoms onset). Informed consent would be supported by a patient (or next to kin) information leaflet in Chinese. A consulted meeting would be developed by a study physician to ensure patients and their families understand the study procedure and consent to participation in the trial [24].

All patients would receive standard care based on the recommendations of AHA/ASA guidelines [25, 26]. For patients with markedly elevated blood pressure, a reasonable goal would be lowering blood pressure by 15 % during the first 24 h. After the first several days, the ideal target blood pressure would be <140/90 mm Hg. Patients with dyslipidemia would be administrated with statin therapy according to low-density lipoprotein cholesterol level. Those patients already taking statins at the time of onset of TIA or ischemic stroke would continue their statin therapy. Patients with diabetes would receive glycemic control according to plasma glucose level. Patients would be suggested early mobilization and several lifestyle modifications, include salt restriction, weight loss, the consumption of a diet rich in fruits, vegetables, and low-fat dairy products, regular aerobic physical activity, smoke quitting and limited alcohol consumption.

The study has been approved by the Medical Ethical Reviewing Committee of the Fourth Military Medical University Medical Center. This clinical trial will be conducted in accordance with the principles laid down by the 18th World Medical Assembly (Helsinki, 1964) and all applicable amendments laid down by the World Medical Assemblies and the International Conference on Harmonization guidelines for Good Clinical Practice.

### Study population

The trial would include (Table 1) male and female patients ≥18 years of age who have an acute MIS or TIA and can be treated with intervention medication within 24 h of symptoms onset. Symptom onset is defined by the “last see normal” principle. The patients could be enrolled if he/she had an acute MIS with NIHSS ≤3 at the time of randomization or had a TIA onset caused by focal brain ischemia with resolution within 24 h of symptom onset and moderate to high risk of stroke recurrence (ABCD^2^ score ≥4 at the time of randomization).

**Table 1:** Inclusion and exclusion criteria

*Inclusion criteria*
✧ Adult subjects (male or female ≥18 years old)
✧ Acute nondisabling ischemic stroke (NIHSS ≤3 at the time of randomization) that can be treated with study drug within 24 h of symptoms onset. Symptom onset is defined by the “last see normal” principle
✧ TIA (neurologic deficit attributed to focal brain ischemia, with resolution of the deficit within 24 h of symptom onset), that can be treated with investigational medication within 24 h of symptoms onset. Symptom onset is defined by the “last see normal” principle
✧ Informed consent signed
*Exclusion criteria*
✧ Diagnosis of hemorrhage or other pathology, such as vascular malformation, tumor, abscess or other major nonischemic brain disease, on baseline head CT or MRI scan
✧ mRS score >2 at randomization (premorbid historical assessment)
✧ NIHSS ≥4 at randomization
✧ Clear indication for anticoagulation (atrial fibrillation, mechanical cardiac valves, deep venous thrombosis, pulmonary embolism or known hypercoagulable state)
✧ Contraindication to investigational medications
✧ Thrombolysis for ischemic stroke within preceding 7 days
✧ History of intracranial hemorrhage
✧ Current treatment (last dose given within 10 days before randomization) with heparin therapy or oral anticoagulation
✧ Gastrointestinal bleed or major surgery within 3 months
✧ Planned or likely revascularization (any angioplasty or vascular surgery) within the next 3 months
✧ TIA or minor stroke induced by angiography or surgery
✧ Severe noncardiovascular comorbidity with life expectancy <3 months
✧ Women of childbearing age not practicing reliable contraception who do not have a documented negative pregnancy test result
✧ Severe renal failure, defined as Glomerular Filtration Rate (GFR) <30 ml/min
✧ Severe hepatic insufficiency (Child-Pugh score B to C)

Patients would be ineligible for the study if they had a diagnosis of hemorrhage or other pathology, such as vascular malformation, tumor, abscess, or other major non-ischemic brain diseases (e.g., multiple sclerosis), on baseline computed tomography (CT) brain scanning or magnetic resonance imaging (MRI) + diffusion weighted imaging (DWI) brain scanning; had modified Rankin Scale (mRS) score >2 at randomization (premorbid historical assessment); had NIHSS ≥4 at randomization; had a clear indication for anticoagulation (presumed cardiac source of embolus, e.g. atrial fibrillation, prosthetic cardiac valves known or suspected endocarditis); had contraindication to investigational medications; are currently treated (last dose given within 10 days before randomization) with heparin therapy or oral anti-coagulation; had history of intracranial hemorrhage; had gastrointestinal bleed or major surgery within 3 months; were planning or likely to undergo revascularization (any angioplasty or vascular surgery) within the next 3 months; had TIA or MIS induced by angiography or surgery; had severe non-cardiovascular comorbidity with life expectancy <3 months; were women of childbearing age not practicing reliable contraception who did not have a documented negative pregnancy test result.

### Study procedures

**Fig. 1:**
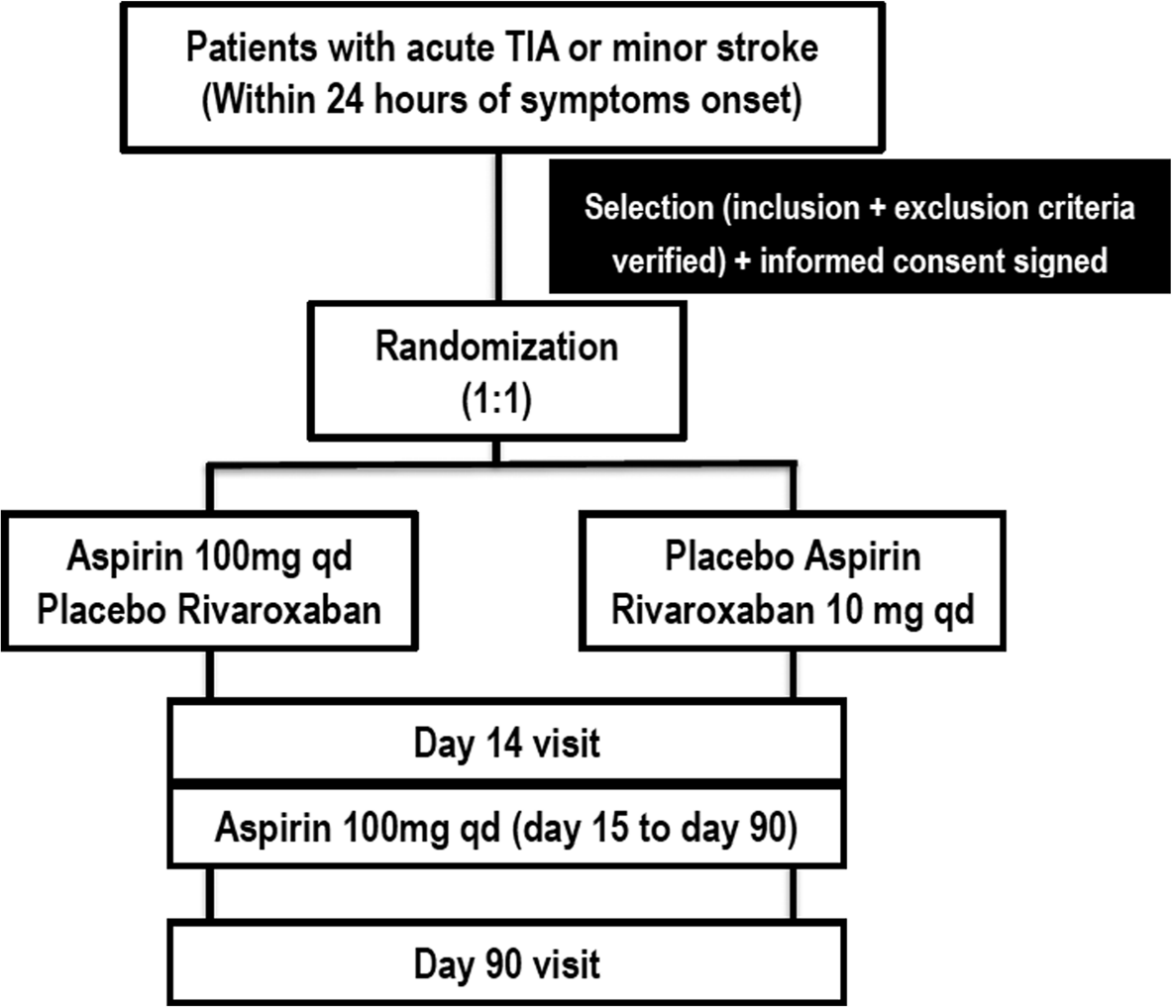
Study flowchart

Figure 1 shows the trial procedures. Participants with suspected TIA or MIS would firstly receive head CT to exclude the intracranial hemorrhage and head MRI + DWI to confirm the area of infarction. A certified, trained and licensed physician investigator would be required to confirm the diagnosis of TIA or MIS and to calculate the ABCD^2^ score for subjects with TIA or NIHSS score for subjects with MIS. Once a standardized, structured interview was performed, the data would be recorded in the case report form for each patient. All clinical data, biological samples and radiological images would be sent to the central study site where a cerebrovascular neurologist would review the data. Demographic, medical, social, and behavioral variables would be determined along with baseline medications. Anthropometry would be conducted using standardized equipment calibrated on a daily basis.

Patients meeting these criteria and offering informed consent would be randomized into 2 arms:

I Receiving a 100-mg dose of aspirin and placebo rivaroxaban
II Receiving a 10-mg dose of rivaroxaban and placebo aspirin

Patients would receive certain study medication from day 1 to day 14. The first dose would be given within 24 h of symptom onset. From day 15 to day 90, all patients would receive 100-mg dose of aspirin as standard antiplatelet therapy. Study visits would be performed on the day of randomization at day 14 and day 90. At randomization and during follow-up visits, the following information would be collected: a neurologic evaluation (mRS and NIHSS); a physical examination, including measurement of weight (kilogram) and vital signs (supine systolic and diastolic blood pressure, heart rate); laboratory tests, a neurological dysfunctions assessment, and concomitant medications and adverse events. For ensuring the safety of patients during the study, the trial should be stopped if the probability that rate of intracranial hemorrhage exceeded 5 % or the rate of other hemorrhagic events is more than 10 %. The progress of the study would be monitored by the data and safety monitoring board for ensuring the high standards of ethics and patient safety.

### Study outcomes

The primary efficacy end point is percentage of patients with any new stroke events (ischemic or hemorrhage), including fatal stroke, at 14 days.

Secondary outcome measures include,

I Percentage of patients at 14 days and 90 days with new clinical vascular events (ischemic stroke/hemorrhagic stroke/TIA/myocardial infarction/vascular death) as a cluster and evaluated individually;
II mRS score, dichotomized at percentage with score 0–2 versus score 3–6, at 14 days and 90 days follow-up;
III Changes in NIHSS scores at 14 days and 90-days follow-up;
IV Efficacy end point would also be analyzed stratified by etiologic subtypes (nonintracranial artery diseases versus intracranial artery diseases), by time randomization (<12 h versus ≥12 h), by qualifying event (TIA versus MIS), and by age (dichotomized at a 5-year cut point closest to median age).

Safety end points include,

I Moderate to severe bleeding event, according to the Global Utilization of Streptokinase and Tissue Plasminogen Activator for occluded Coronary Arteries definition, including fatal bleeding, intracranial hemorrhage and other hemorrhagic events required transfusion of blood, even causing hemodynamic compromise requiring intervention at 90 days;
II Total mortality at 14-days follow-up;
III Adverse events/severe adverse events reported by the investigators in 14-days follow-up.

### Randomization, allocation concealment and blinding

Subject numbers would be assigned sequentially as each patient enters the study. After receiving an informed consent, patients fulfilling the inclusion criteria would be randomized by computer-generated random numbers (Microsoft Excel 2010, Redmond, WA, USA) at statistics research office, Fourth Military Medical University. A clinical research associate would make opaque blinded envelopes (with consecutive numbers) and deliver them to each participating center. Random allocation would be performed within 24 h after a patient enrolled. The block size and treatment-assignment table would not be available to the researchers until the end of the trial.

The study medication would be stored under the conditions specified on the label in a locked, safe area of the pharmacy department to prevent unauthorized access. Both the study medication and the placebo would be indistinguishable; they would be manufactured by the same company and similar in appearance, organoleptic characteristics, and presentation.

In the event of emergency, an investigator (HJL) and a clinical pharmacist (JYY) would decide whether it’s necessary to unblind the subject’s treatment assignment using the unblinding envelopes provided to the hospital. If unblinding was necessary, the investigator must record the reason for unblinding, as well as the date and time of the event. In each participating center, an independent researcher would be assigned to collect the trial paper and electronic records. In the central trial coordinating office, an independent research assistant (ZZR) would have access to all unblinded data and maintain confidentiality of patient records.

### Statistical considerations

The primary null hypothesis of this trial is as follows: in patients with TIA or MIS treated with aspirin 100 mg per day, there would be no difference in 14-days risk of stroke (ischemic or hemorrhagic) in those treated with a 14-days regimen of rivaroxaban 10 mg per day, when therapy were initiated within 24 h of symptom onset.

The minimum necessary sample size in the trial is established by the requirement to detect the smallest expected clinically meaningful treatment difference comparing the treatment with placebo. According to the results of previous clinical studies [4, 5], we speculated the 14-day risk of stroke recurrence in the placebo (aspirin) group was about 10 % among TIA or MIS patients treated with aspirin within 24 h of symptom onset. The results from our preliminary study were used to calculate the sample size (unpublished data). A relative risk reduction of 28 % (relative risk with addition of rivaroxaban is 0.72) was the smallest difference we would attempt to detect. The following equation was used for calculating the sample size:

$$ \mathrm{n}=\frac{1}{2}{\left(\frac{u_{\alpha }+{u}_{\beta }}{{ \sin}^{-1}\sqrt{p1}-{ \sin}^{-1}\sqrt{p2}}\right)}^2 $$

With a two-sided 5 % significance level (α) and a 90 % power (1-β), *p*1 of 0.10, *p*2 of 0.072, the minimal sample size per group was estimated to be 2095 patients. About 4400 participants in total 2 groups was a final estimated sample size with 5 % dropouts (medication nonadherence). Missing values would be remained missing, and patients would be censored at their last follow-up assessment (time of clinical event, end of study, or last visit before loss to follow-up).

All analyses would be intention to treat. In these analyses we would compare conventional-dose of rivaroxaban versus aspirin. Kaplan-Meier estimates of the cumulative risk of a stroke (ischemic or hemorrhagic) event during a 14 days’ follow-up, with hazard ratios and 95 % confidence intervals calculated using Cox proportional hazards methods and the log-rank test to evaluate the treatment effect would be reported. The Chi-square test of association would be used to compare groups at baseline as appropriate. Logistic regression would be used to determine the significance of the results obtained. All statistics would be two-sided with *p* <0.05 considered significant.

### Study organization

The progress of the study would be monitored by the Data and Safety Monitoring Board (DSMB) to ensure the high standards of ethics and patient safety. The DSMB would monitor the trial via scheduled and unscheduled reviews, supervise stopping rules and unmasking, and maintain the confidentiality of internal discussions and validity of the reports.

An Executive Committee meeting would be organized to make major decisions. Quality control inspectors would be responsible for inspecting the quality of research sites and study data periodically, supervising any quality problem during the trial, and reporting to the Executive Committee. The members of the Steering Committee, including two project directors, would be members of the Executive Committee and would convene monthly (teleconferences or physical meetings) to review the status of the trial and available blinded data; they would take appropriate action regarding the conduct of the study.

An Adjudication Committee charter including membership, role, and responsibilities would be approved before the start of the trial by the Adjudication Committee and the Executive Committee. This committee would be composed of Academic Members, including an independent statistician, who were not otherwise participating in the trial.

## Discussion

TIA and MIS have commonly been referred as non-disabling cerebrovascular events and often portend disabling stroke. Although previous study showed that rapid assessment and early treatment after a non-disabling cerebrovascular event resulted in a much lower risk of recurrent stroke [25], few established or effective therapies were used in clinical work. Aspirin is the current standard antiplatelet therapy to prevent recurrent stroke in patients with acute cerebrovascular event, but the effect is modest, and moreover weakened by a small increased risk of intracranial hemorrhage. Traditional anticoagulant, if administrated immediately after acute ischemic stroke, could decrease risk of recurrent stroke compared to patients untreated or received antiplatelet agent [12, 27]. However, since increased rate of bleeding complications, the usefulness of anticoagulation is still in dispute.

There’re few clinical trials to test the effect of new oral anticoagulants on non-disabling cerebrovascular events, even though these agents seem to be safe and effective. Rivaroxaban (a direct factor Xa inhibitor), one of the new anticoagulants, has received attention widely. Recently it has been confirmed effective in prevention and treatment of DVT, VTE and pulmonary embolism, and showed improved outcome in patients with atrial fibrillation and with acute coronary syndrome [20–22].

However, effects of rivaroxaban for prevention of the early recurrent risk after TIA and acute MIS are still unsettled. Anticoagulant therapy, with the new drug rivaroxaban, might prevent more cerebrovascular events with an acceptable risk profile after TIA or MIS compared with mono-antiplatelet therapy, thus would be helpful to improve the outcome of the disease.

We designed TRACE trial as a randomized, double-blind, multicenter, controlled clinical trial in China. We would assess the hypothesis that a 14-days rivaroxaban regimen followed to aspirin administration is superior to aspirin alone for the treatment of high-risk patients with acute nondisabling cerebrovascular event.

## Trail status

The trial was registered at Clinicaltrials.org and the study is open for recruitment.

## Abbreviations

CT: Computed tomography
DWI: Diffusion weighted imaging
DVT: Deep-vein thrombosis
MIS: Minor ischemic stroke
MRI: Magnetic resonance imaging
mRS: Modified Rankin Scale
NIHSS: National Institutes of Health Stroke Scale
TIA: Transient ischemic attack
t-PA: Tissue plasminogen activator
VTE: Venous thromboembolism

## References

[1] Brott T, Adams HP, Olinger CP, Marler JR, Barsan WG, Biller J, Spilker J, Holleran R, Eberle R, Hertzberg V (1989). Measurements of acute cerebral infarction: a clinical examination scale. Stroke.

[2] Giles MF, Rothwell PM (2007). Substantial underestimation of the need for outpatient services for TIA and minor stroke. Age Ageing.

[3] Edlow JA, Kim S, Pelletier AJ, Camargo CA (2006). National study on emergency department visits for transient ischemic attack, 1992–2001. Acad Emerg Med.

[4] Coull AJ, Lovett JK, Rothwell PM, Oxford Vascular S (2004). Population based study of early risk of stroke after transient ischaemic attack or minor stroke: implications for public education and organisation of services. BMJ.

[5] Johnston SC, Gress DR, Browner WS, Sidney S (2000). Short-term prognosis after emergency department diagnosis of TIA. JAMA.

[6] Rothwell PM, Buchan A, Johnston SC (2006). Recent advances in management of transient ischaemic attacks and minor ischaemic strokes. Lancet Neurol.

[7] Graham GD (2003). Tissue plasminogen activator for acute ischemic stroke in clinical practice: a meta-analysis of safety data. Stroke.

[8] Kleindorfer D, Kissela B, Schneider A, Woo D, Khoury J, Miller R, Alwell K, Gebel J, Szaflarski J, Pancioli A (2004). Eligibility for recombinant tissue plasminogen activator in acute ischemic stroke: a population-based study. Stroke.

[9] De Keyser J, Gdovinova Z, Uyttenboogaart M, Vroomen PC, Luijckx GJ (2007). Intravenous alteplase for stroke: beyond the guidelines and in particular clinical situations. Stroke.

[10] Smith EE, Abdullah AR, Petkovska I, Rosenthal E, Koroshetz WJ, Schwamm LH (2005). Poor outcomes in patients who do not receive intravenous tissue plasminogen activator because of mild or improving ischemic stroke. Stroke.

[11] Chen ZM, Group CC (1997). CAST: randomised placebo-controlled trial of early aspirin use in 20,000 patients with acute ischaemic stroke. CAST (Chinese Acute Stroke Trial) Collaborative Group. Lancet.

[12] International Stroke Trial Collaborative Group (1997). The International Stroke Trial (IST): a randomised trial of aspirin, subcutaneous heparin, both, or neither among 19435 patients with acute ischaemic stroke. International Stroke Trial Collaborative Group. Lancet.

[13] Bhatt DL, Fox KA, Hacke W, Berger PB, Black HR, Boden WE, Cacoub P, Cohen EA, Creager MA, Easton JD (2006). Clopidogrel and aspirin versus aspirin alone for the prevention of atherothrombotic events. N Engl J Med.

[14] Benavente OR, Hart RG, McClure LA, Szychowski JM, Coffey CS, Pearce LA, S.P.S. Investigators (2012). Effects of clopidogrel added to aspirin in patients with recent lacunar stroke. N Engl J Med.

[15] Wang Y, Wang Y, Zhao X, Liu L, Wang D, Wang C, Wang C, Li H, Meng X, Cui L (2013). Clopidogrel with aspirin in acute minor stroke or transient ischemic attack. N Engl J Med.

[16] Esprit (2003). Oral anticoagulation in patients after cerebral ischemia of arterial origin and risk of intracranial hemorrhage. Stroke.

[17] Chimowitz MI, Lynn MJ, Howlett-Smith H, Stern BJ, Hertzberg VS, Frankel MR, Levine SR, Chaturvedi S, Kasner SE, Benesch CG (2005). Comparison of warfarin and aspirin for symptomatic intracranial arterial stenosis. N Engl J Med.

[18] Mohr JP, Thompson JL, Lazar RM, Levin B, Sacco RL, Furie KL, Kistler JP, Albers GW, Pettigrew LC, Adams HP (2001). A comparison of warfarin and aspirin for the prevention of recurrent ischemic stroke. N Engl J Med.

[19] Sacco RL, Prabhakaran S, Thompson JL, Murphy A, Sciacca RR, Levin B, Mohr JP, Investigators W (2006). Comparison of warfarin versus aspirin for the prevention of recurrent stroke or death: subgroup analyses from the Warfarin-Aspirin Recurrent Stroke Study. Cerebrovasc Dis.

[20] Furie KL, Goldstein LB, Albers GW, Khatri P, Neyens R, Turakhia MP, Turan TN, Wood KA (2012). Oral antithrombotic agents for the prevention of stroke in nonvalvular atrial fibrillation: a science advisory for healthcare professionals from the American Heart Association/American Stroke Association. Stroke.

[21] Patel MR, Mahaffey KW, Garg J, Pan G, Singer DE, Hacke W, Breithardt G, Halperin JL, Hankey GJ, Piccini JP (2011). Rivaroxaban versus warfarin in nonvalvular atrial fibrillation. N Engl J Med.

[22] Granger CB, Alexander JH, McMurray JJ, Lopes RD, Hylek EM, Hanna M, Al-Khalidi HR, Ansell J, Atar D, Avezum A (2011). Apixaban versus warfarin in patients with atrial fibrillation. N Engl J Med.

[23] Harrington AR, Armstrong EP, Nolan PE, Malone DC (2013). Cost-effectiveness of apixaban, dabigatran, rivaroxaban, and warfarin for stroke prevention in atrial fibrillation. Stroke.

[24] Allmark P, Mason S (2006). Improving the quality of consent to randomised controlled trials by using continuous consent and clinician training in the consent process. J Med Ethics.

[25] Jauch EC, Saver JL, Adams HP, Bruno A, Connors JJ, Demaerschalk BM, Khatri P, McMullan PW, Qureshi AI, Rosenfield K (2013). Guidelines for the early management of patients with acute ischemic stroke: a guideline for healthcare professionals from the American Heart Association/American Stroke Association. Stroke.

[26] Kernan WN, Ovbiagele B, Black HR, Bravata DM, Chimowitz MI, Ezekowitz MD, Fang MC, Fisher M, Furie KL, Heck DV (2014). Guidelines for the prevention of stroke in patients with stroke and transient ischemic attack: a guideline for healthcare professionals from the American Heart Association/American Stroke Association. Stroke.

[27] The Publications Committee for the Trial of ORG 10172 in Acute Stroke Treatment (TOAST) Investigators (1998). Low molecular weight heparinoid, ORG 10172 (danaparoid), and outcome after acute ischemic stroke: a randomized controlled trial. JAMA.

